# Curcumin Blocks Cytotoxicity of Enteroaggregative and Enteropathogenic *Escherichia coli* by Blocking Pet and EspC Proteolytic Release From Bacterial Outer Membrane

**DOI:** 10.3389/fcimb.2019.00334

**Published:** 2019-09-25

**Authors:** Javier I. Sanchez-Villamil, Fernando Navarro-Garcia, Araceli Castillo-Romero, Filiberto Gutierrez-Gutierrez, Daniel Tapia, Gabriela Tapia-Pastrana

**Affiliations:** ^1^Department of Cell Biology, Centro de Investigación y de Estudios Avanzados del IPN (CINVESTAV-IPN), Mexico City, Mexico; ^2^Department of Microbiology and Pathology, Centro Universitario de Ciencias de la Salud, Universidad de Guadalajara, Guadalajara, Mexico; ^3^Department of Chemistry, Centro Universitario de Ciencias Exactas e Ingenierías, Universidad de Guadalajara, Guadalajara, Mexico; ^4^Department of Microbiology and Immunology, University of Texas Medical Branch, Galveston, TX, United States; ^5^Laboratory of Biomedical Investigation, Hospital Regional de Alta Especialidad de Oaxaca, San Bartolo Coyotepec, Mexico

**Keywords:** *Escherichia coli*, pathogenic *E. coli*, autotransporter, SPATE, beta-barrel, antimicrobial agent, curcumin, cytoskeletal disruption

## Abstract

Pet and EspC are toxins secreted by enteroaggregative (EAEC) and enteropathogenic (EPEC) diarrheagenic *Escherichia coli* pathotypes, respectively. Both toxins are members of the Serine Protease Autotransporters of *Enterobacteriaceae* (SPATEs) family. Pet and EspC are important virulence factors that produce cytotoxic and enterotoxic effects on enterocytes. Here, we evaluated the effect of curcumin, a polyphenolic compound obtained from the rhizomes of *Curcuma longa* L. (Zingiberaceae) on the secretion and cytotoxic effects of Pet and EspC proteins. We found that curcumin prevents Pet and EspC secretion without affecting bacterial growth or the expression of *pet* and *espC*. Our results show that curcumin affects the release of these SPATEs from the translocation domain, thereby affecting the pathogenesis of EAEC and EPEC. Curcumin-treated EAEC and EPEC did not induce significant cell damage like the ability to disrupt the actin cytoskeleton, without affecting their characteristic adherence patterns on epithelial cells. A molecular model of docking predicted that curcumin interacts with the determinant residues Asp_1018_-Asp_1019_ and Asp_1029_-Asp_1030_ of the translocation domain required for the release of Pet and EspC, respectively. Consequently, curcumin blocks Pet and EspC cytotoxicity on epithelial cells by preventing their release from the outer membrane.

## Introduction

Diarrheagenic diseases are a significant public health concern as well as an important source of morbidity and mortality. In 2016, diarrheal diseases were responsible for more than 1.6 million deaths worldwide, of which ~27% were in children under 5 years of age (Troeger et al., 2018). The primary etiological agents of diarrheal diseases include Rotavirus, Calicivirus, and diarrheagenic *Escherichia coli*. The distinct diarrheagenic *E. coli* are categorized into separate pathotypes according to their broad spectrum of disease associated with different clinical symptoms as a result of the combination of several virulence factors and pathogenic mechanisms. These different pathogenic organisms have been classified into six different pathotypes (Croxen et al., 2013). Among these, enteroaggregative (EAEC) and enteropathogenic (EPEC) are among the most commonly associated with disease and are responsible for nearly 30–40% of diarrheal episodes in low to middle-income countries (Ramya Raghavan et al., 2017). Both EAEC and EPEC secrete the autotransporter (AT) proteins acting as toxins, including the plasmid-encoded toxin (Pet) of 104 kDa (Eslava et al., 1998; Navarro-Garcia et al., 1999), and EPEC-secreted protein C (EspC) of 110 kDa (Stein et al., 1996; Navarro-Garcia et al., 2004), respectively. Both Pet and EspC are toxins belonging to the subfamily of Serine Protease Autotransporters of *Enterobacteriaceae* (SPATEs). AT proteins from the SPATE subfamily are secreted by bacteria using a Type V secretion system and have several common structural characteristics (Dautin, 2010). All AT proteins contain an N-terminal signal sequence, a passenger domain which commonly encodes a virulence function that is secreted into the extracellular milieu, and a translocation domain (β-barrel) which resides on the outer membrane, from which the mature passenger domain is released for its secretion (Jose et al., 1995; Henderson et al., 2004). Despite their structural similarities, the AT proteins Pet and EspC have different interactions with host cells (Navarro-Garcia et al., 2010). Nonetheless, both toxins produce cytotoxic effects that are strictly dependent on the internalization in the cytoplasm of epithelial cells. The catalytic activity of SPATES, such as Pet and EspC, is found within the passenger domain and is responsible for cleaving various substrates proteins such as fodrin, which is involved in actin crosslinking and is essential for the maintenance of the cytoskeleton architecture (Dautin, 2010).

Notwithstanding the progress in the development of antimicrobial agents, there is a need to elucidate alternative antimicrobial compounds as a result of the rise of multidrug-resistant bacteria (Wise et al., 1998). The increase in antibiotic resistance has driven the exploration to identify and evaluate alternative, and often, natural compounds with antimicrobial activities. Curcumin, a polyphenolic compound obtained from the rhizomes of *Curcuma longa* L. (Zingiberaceae), has been utilized as a spice, preservative, and natural food coloring agent (Ammon and Wahl, 1991). Different studies have demonstrated the broad spectrum of potential biological activities for curcumin, such as its antimicrobial, antiviral, and antifungal properties affecting the physiology of these organisms through various mechanisms (Moghadamtousi et al., 2014). In bacteria such as uropathogenic *E. coli*, and *Streptococcus mutans*, it has been demonstrated that curcumin affects the formation of biofilm, an important mechanism for bacterial adherence, aggregation, and resistance to antibiotics (Packiavathy et al., 2014; Li et al., 2018). Curcumin has also been associated with disrupting the function of virulence factors such as the flagellum of *Salmonella enterica* serovar Typhimurium (Marathe et al., 2016), the cellular permeability of *E. coli* and *Staphylococcus aureus* (Tyagi et al., 2015), as well as influencing the cell division process of *B. subtilis* (Rai et al., 2008). A synergistic antibacterial effect has also been attributed to curcumin when given with different antibiotics against different strains of *Staphylococcus aureus* (Teow and Ali, 2015). Using a weaning pig as an animal model, curcumin has previously been reported to confer a protective effect in animals challenged with enterotoxigenic *E. coli* (ETEC) (Xun et al., 2015). Therefore, this study aimed to determine the role of curcumin in the pathogenesis of SPATE-producing diarrheagenic *E. coli* by evaluating the inhibition of cytotoxic effects induced by SPATEs such as Pet and EspC, which are secreted by EAEC and EPEC, respectively. This study proposes a molecular mechanism whereby curcumin inhibits the secretion of SPATEs (Pet and EspC) by preventing their release from the outer membrane of these two bacterial pathogens, thereby blocking a key component of their mechanism of pathogenesis.

## Materials and Methods

### Bacterial Strains

Diarrheagenic *E. coli* used in this study, EPEC (E2348/69) (Levine et al., 1978) and EAEC (042) (Nataro et al., 1995), were routinely grown aerobically at 37°C in Luria-Bertani (LB) broth. Before cell infection, the overnight cultures were activated in DMEM medium without fetal bovine serum (FBS) for the induction of virulence factors (Rosenshine et al., 1996) and without antibiotics. Bacterial cultures were incubated for 2 h at 37°C under static growth.

### Bacterial Growth and SPATEs Secretion

Overnight bacterial cultures of EAEC and EPEC were diluted to an initial optical density (OD_600nm_) of 0.05. Curcumin was diluted in Dimethyl Sulfoxide (DMSO) (Biobasic-Canada Inc. CAS67-68-5) to ensure complete solubilization to 10 mg/mL. The strains EAEC and EPEC (10 mL) were incubated using different concentrations of curcumin (Curcuma longa ≥65%, C1386 SIGMA) (8, 16, 32, 64 μg/mL) for 2 and 4 h at 37°C and bacterial growth was evaluated by optical density (OD_600nm_) using a spectrophotometer (BioPhotometer plus Eppendorf). For SPATEs secretion, the bacterial cultures were centrifuged at 2,500 × g for 20 min, supernatants were filtered through a 0.45 μm filter, and the proteins in the supernatant were precipitated using absolute ethanol overnight at −20°C. After precipitation, the supernatants were centrifuged at 14,000 × g for 30 min at 4°C, dried, and resuspended in Tris-HCl pH 8.8 and then quantified using a micro-Bradford (Bradford, 1976). All samples were analyzed by Western-Blot using anti-Pet and anti-EspC passenger domain specific antibodies.

### Periplasmic and Outer Membrane Fractionations

Bacterial cultures of EAEC and EPEC (100 mL) were incubated with curcumin using a final concentration of 16 μg/mL for 2 h at 37°C. Cultures were harvested by centrifugation at 2,500 × g for 20 min at 4°C. Bacterial pellets were suspended in 10 mL of 30 mM Tris-HCl with 20% sucrose and 1 mM EDTA (pH 8.0), incubated for 10 min, and centrifuged at 8,000 × g for 10 min. Bacterial pellets were then resuspended in 500 μl of 5 mM MgSO_4_ and shaken for 10 min on ice. The suspension was centrifuged at 8,000 × g for 10 min at 4°C, and the supernatants containing the periplasmic fractions were collected and stored at −20°C in presence of complete™ Protease Inhibitor Cocktail (1 ×) (Roche, USA). The quality of the purified periplasmic fractions was tested by Western blot assay to detect β-lactamase, a periplasmic protein, using a specific anti-β-lactamase antibody (Chemicon, Temecula, CA). Primary polyclonal rabbit-anti-GroEL antibody (Abcam) was used to detect cytoplasmic fraction (1:1,000). Outer membrane proteins were obtained by using a Sarkosyl-extraction method, as previously described by Klingman and Murphy (1994). To extract the outer membrane components, the bacterial cultures were grown as indicated above. Cultures were harvested by centrifugation at 7,000 × g for 20 min at 4°C. The pellet was resuspended in 10 mL of 10 mM HEPES (pH 7.4), and the cells were lysed by sonication for 60 s ON and 60 s OFF five times on ice-water using a Soniprep sonicator at 50% amplitude. Unbroken cells were removed by centrifugation at 2,500 × g for 30 min at 4°C. The supernatant was centrifuged at 100,000 × g for 1 h at 4°C. The pellet was resuspended in 100 mM Tris-HCl (pH 7.2) with 1% Sarkosyl and incubated at room temperature for 30 min. After that, the suspension was centrifuged at 100,000 × g for 1 h at 4°C. The pellet was resuspended in 10 mM HEPES (pH 7.4). This fraction was also used to detect the translocation-domains from Pet and EspC, using a mouse anti-β-barrel translocation unit antibody. Antibodies recognizing the translocation domain (β-barrel) of Pet were generated by immunizing mice with purified translocation domain protein extracted from the outer membrane fraction separated by SDS-PAGE electrophoresis. The corresponding band (30 kDa) of the translocation domain was excised, purified by electrolution, and pooled fractions were used to immunize mice intraperitoneally with 50 μg of total protein. Mice immunized with the translocation domain were exsanguinated and sera was separated by centrifugation (5,000 × g) for 5 min. These antibodies also recognized the translocation domain of EspC due to their high sequence homology.

### SDS-PAGE and Western Blotting Assays

Supernatant, periplasmic, and outer membrane fractions from bacterial cells were quantified by the micro-Bradford method and resolved by SDS-PAGE on 12% polyacrylamide gels. The proteins were transferred to nitrocellulose membranes for western blot analysis. Immunoblotting was performed using rabbit primary antibodies against Pet and EspC passenger domains (1:500 dilutions), and a mouse primary antibody that recognizes the translocation domain (β-barrel) (1:250 dilution). A Horseradish peroxidase (HRP)-conjugated goat anti-rabbit IgG secondary antibody or an anti-mouse IgG2a secondary antibody (Zymed, Grand Island, NY) was used according to the manufacturer's instructions.

### RT-PCR Assay

#### Bacterial RNA Extraction

Bacterial cultures from (EAEC and EPEC) treated with curcumin (8, 16, 32, 64 μg/mL) or DMSO were used to extract RNA by using RNeasy Mini extraction (Qiagen) following manufacturers recommendations. DNA contamination was completely removed using RNase-Free DNase according to the manufacturer's instructions (QIAGEN). The integrity of total RNA was initially assessed by visualization of the 23S/16S banding pattern using 1.2% agarose gel electrophoresis, the gel was treated with ethidium bromide and documented by capture system Benchtop 2UV Transilluminator UVP.

#### Expression of Genes Coding for Secreted Proteins

The expression *pet* and *espC* was evaluated by monitoring the corresponding transcript levels after bacteria were exposed to 8, 16, 32, or 64 μg/mL of curcumin or the vehicle control (DMSO). We used 2 μg of total RNA for cDNA synthesis by reverse transcription using RT-PCR kit (TaKaRa). The specific primers for *pet* and *espC* sequences were as follows: forward *pet* 5′-ggcacagaataaaggggtgttt-3′ and reverse 5′-cctcttgtttccacgacatac-3′ forward *espC* 5′-tagtgcagtgcagaaagcagtt-3′, and reverse 5′-agttttcctgttgctgtatgcc-3′. Reverse transcriptase polymerase chain reaction (RT-PCR) products were obtained by PCR amplification for 30 cycles as follows: 94°C for 1.5 min, 60°C for 1.5 min and 72°C for 1.5 min, with an initial denaturation step of 94°C for 5 min and final extension step of 72°C for 10 min. The PCR products were analyzed by electrophoresis on 1.5% agarose gels containing ethidium bromide and documented by capture system Benchtop 2UV Transilluminator UVP.

### Cell Infection Assay

HEp-2 human laryngeal carcinoma cell line (ATCC CCL-23) was routinely grown in DMEM (Corning, USA) supplemented with 0.1 mM non-essential amino acids, 100 U of penicillin/mL, 100 μg of streptomycin/mL, and 10% FBS (Premium, Corning, USA). Cells were incubated at 37°C in a humidified incubator at 5% CO_2_. Cells were routinely harvested with 10 mM EDTA and 0.25% trypsin (Corning, USA) in PBS (pH 7.4), resuspended in the supplemented DMEM, and incubated at 37°C. For analysis by confocal microscopy, the HEp-2 cells were cultured in Labtek slides (VWR, Batavia, IL, USA) and incubated for 48 h to reach a 90% of confluence. Bacterial cultures were incubated for 2 h in the presence or absence of curcumin (16 μg/mL), then washed with DMEM prior to the infection of HEp-2 cells. Cells were infected with the corresponding inoculum using a multiplicity of infection (MOI) of 10 and incubated for an additional 2 h. After infection, cells were washed and fixed with 4% paraformaldehyde-PBS. Polymerized actin was detected by staining with tetramethyl rhodamine isothiocyanate-phalloidin (Molecular Probes-Invitrogen, Carlsbad, CA, USA). DNA from nuclei and bacteria were detected using TO-PRO-3 (Molecular Probes-Invitrogen, Carlsbad, CA, USA). Pet and EspC proteins were detected by immunofluorescence using polyclonal anti-Pet or anti-EspC (passenger domains) antibodies using a dilution of 1:50, followed by biotin-SP-conjugated goat anti-rabbit IgG and DTAF-conjugated streptavidin. Detailed descriptions of the generation of antibodies used in this study are described elsewhere (Navarro-Garcia et al., 2004, 2007). Slides were mounted with VectaShield (Vector Laboratories, Burlingame, CA, USA) covered with glass coverslips, and analyzed using a Leica Confocal Microscope TCS SP8 (Leica Microsystems, Wetzlar, Germany) and Leica LAS AF lite software.

### Homology Modeling

The three-dimensional (3D) structures of the translocation domain (β-barrel) of the autotransporter proteins Pet and EspC were constructed by the homology modeling principle. In this study, we carried out the homology modeling process with SWISS-MODEL server (Biasini et al., 2014), using the amino acid sequences of EspC (GenBank accession no. AAC44731.1) and Pet (GenBank accession no. SJK83553.1) and using the extracellular serine protease EspP (PDB: 3SLJ) crystallographic structure as template (Barnard et al., 2012). Optimization of the hydrogen bonding network and the atomic level energy minimization of the β-barrel models generated were performed using the WHAT IF Web Interface (Vriend, 1990). The global quality, stereochemical values, and non-bonded interactions of models were tested using the Qualitative Model Energy Analysis (QMEANscore6) (Benkert et al., 2009), RAMPAGE (Lovell et al., 2003), ERRAT (Colovos and Yeates, 1993), and VERIFY3D (Eisenberg et al., 1997).

### Molecular Docking

Possible binding sites to the translocation domain of EspC and Pet proteins were evaluated to identify the most important ligand protein interactions. The curcumin structure was built and submitted to a geometry optimization protocol using the Maestro software 2017-1 (Schrödinger, LLS, New York, NY, USA) (Schrödinger, 2015). Polar hydrogens, Gasteiger-Marsili empirical atomic partial charges (Gasteiger and Marsili, 1978) and the AutoDock atom types of the curcumin and the autotransporter structures were computated employing the MGLTools v1.5.6 package (Morris et al., 2009). The overall combined bindings of curcumin with EspC or Pet were evaluated, first using a grid box covering the Pet and EspC translocation domains (β-barrel) (26 × 50 × 50 Å^3^) to assess the specific curcumin-translocation domain binding sites. Next, to confirm the correct position, a second docking on the site with the greatest cluster was performed using a grid box measuring 22 × 32 × 26 Å^3^. A total of 10 binding modes were generated with an exhaustiveness value of 150. The lowest binding free energy conformation and the related binding modes (RMSD <2 Å) were selected for the analysis. The results were visualized with the MAESTRO software (Schrödinger, 2015).

## Results

### Curcumin Treatment Does Not Affect Bacterial Growth of Diarrheagenic *Escherichia coli* (EAEC and EPEC)

**Figure 1:**
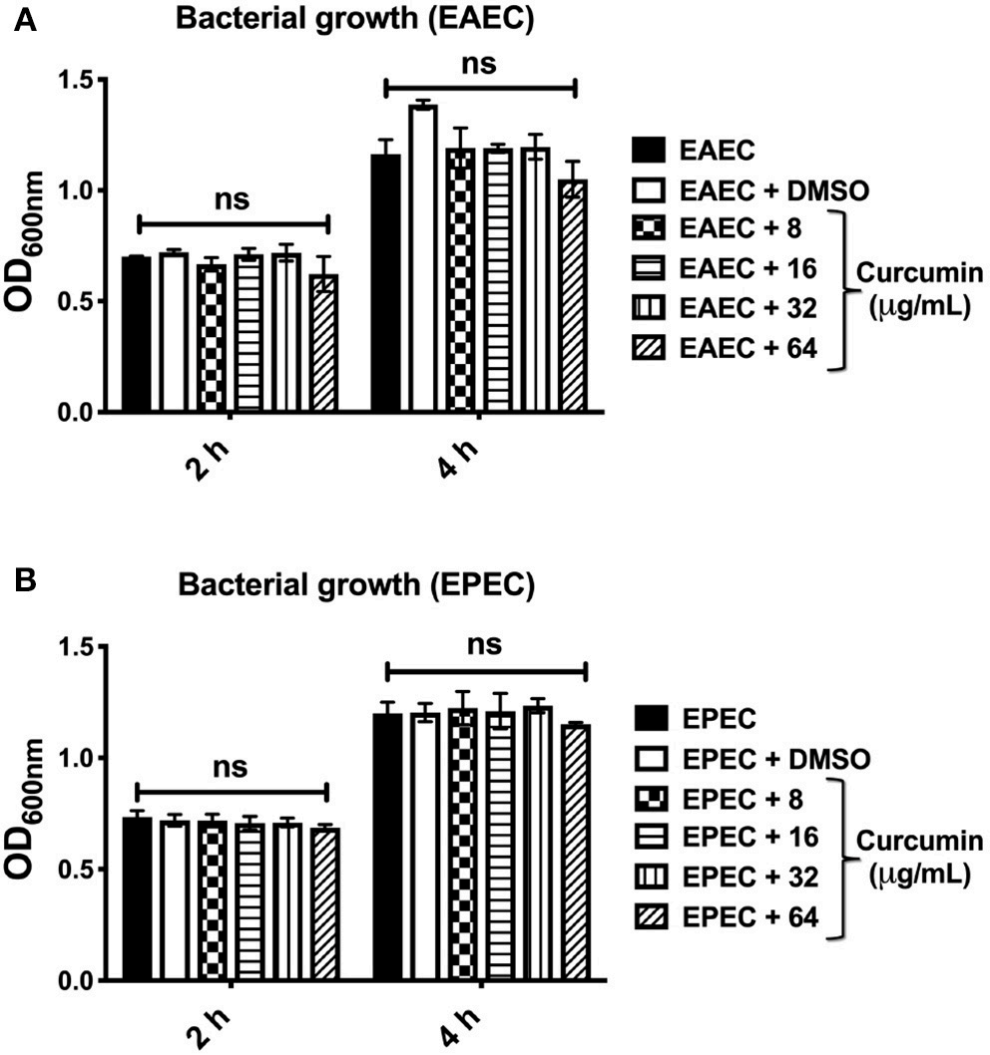
Curcumin has no toxic effect on EAEC and EPEC bacterial strains. Bacterial strains EAEC 042 **(A)** and EPEC E2348/69 **(B)** were grown in LB broth to an initial optical density of 0.05 (OD_600nm_), when the bacteria reached an exponential growth phase, different concentrations of curcumin were added (8, 16, 32, 64 μg/mL) for 2 and 4 h at 37°C at 150 rpm. Bacterial strains without curcumin (black bars) or with vehicle DMSO (white bars) served as internal control. The values in all the graphs represent means ± SEM for at least three independent experiments. Statistical significance was determined by using a two-way ANOVA test followed by a Tukey *post-hoc* test, no significance (ns) (*p* > 0.05).

Curcumin has previously been demonstrated to have antimicrobial properties *in vitro* against several Gram-positive and Gram-negative bacteria (Rai et al., 2008; Luer et al., 2012; Mun et al., 2013; Betts and Wareham, 2014). In order to evaluate and compare the cytotoxicity of curcumin against two diarrheagenic *E. coli* strains (EAEC and EPEC), we evaluated the effect of this compound on bacterial growth. Bacterial cultures (initial OD_600nm_ of 0.05) were incubated with four different concentrations of curcumin (8, 16, 32, 64 μg/mL) for 2 and 4 h in LB broth. However, curcumin treatment did not affect the growth of either EAEC (Figure 1A) or EPEC (Figure 1B), given that there were no significant differences compared with Dimethyl sulfoxide (DMSO)-treated, or untreated bacterial cultures. These results are consistent with those published previously which show that high concentrations of curcumin (100 and 375 μg/mL, respectively), does not affect the growth of two different *E. coli* strains (uropathogenic and Migula) (Packiavathy et al., 2014; Gunes et al., 2016).

### Curcumin Decreases the Secretion of SPATEs Such as Pet (EAEC) and EspC (EPEC) in a Concentration- and Time-Dependent Manner

**Figure 2:**
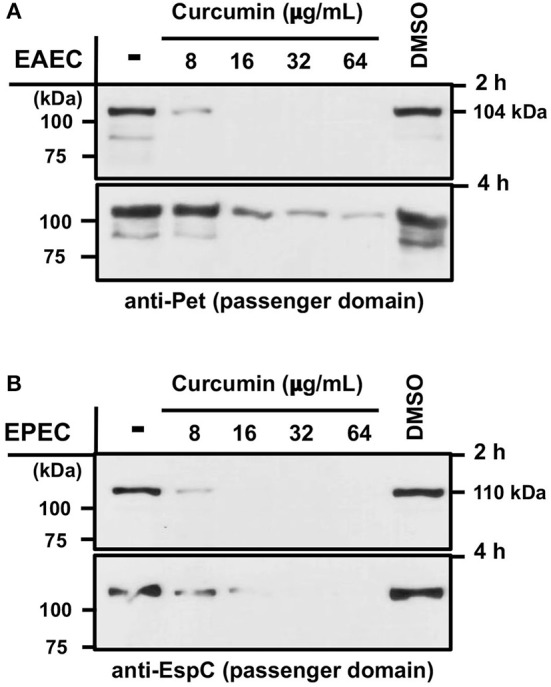
Curcumin inhibits the secretion of SPATEs Pet and EspC (from EAEC and EPEC, respectively) in a concentration- and time-dependent manner. Bacterial cultures of EAEC 042 and EPEC E2348/69 were grown using different curcumin concentrations of (8, 16, 32, 64 μg/mL) for 2 and 4 h at 37°C at 150 rpm. Bacteria were pelleted and the culture supernatants were precipitated for proteins collection (see section Materials and Methods). Protein bands were analyzed by Western-blot using a specific antibody for Pet **(A)** and EspC **(B)** passenger domains, followed by HRP-conjugated goat anti-rabbit IgG secondary antibody. As controls, bacterial strains without curcumin or with vehicle (DMSO) were used.

Given that curcumin treatment did not alter the growth of either EAEC or EPEC, we evaluated the secretion of two key virulence factors (Pet from EAEC and EspC from EPEC) involved in the cytotoxic effects induced by these two *E. coli* pathotypes. As mention before, Pet and EspC belong to the class 1 of Serine Protease Autotransporters of *Enterobacteriaceae* (SPATEs) which use a type 5 secretion system (T5SS) for export into the extracellular milieu. In order to evaluate whether curcumin has an effect on Pet or EspC secretion, bacterial cultures of EAEC, and EPEC were incubated with curcumin (8, 16, 32, 64 μg/mL) for 2 and 4 h. After incubation in the presence of curcumin, culture supernatants were collected by centrifugation, and the secretion of Pet and EspC (passenger domains) was evaluated by Western-blot (WB) analysis. Two-hours after curcumin treatment, the secretion of Pet (Figure 2A, upper panel) and EspC (Figure 2B, upper panel) were completely inhibited starting with 16 μg/mL, and higher concentrations (32 and 64 μg/mL), although only partially inhibited at the lowest concentration (8 μg/mL). After 4 h of curcumin treatment (8 μg/mL), the secretion of Pet was recovered at levels comparable with those of untreated bacteria, but this secretion gradually decreased in a curcumin concentration-dependent manner (Figure 2A, lower panel). Contrastingly, EspC secretion at 4 h was completely inhibited at the highest concentration, although only partially at the lowest concentration of curcumin (Figure 2B, lower panel). No differences in the secretion of Pet or EspC were seen in bacteria treated with either Dimethyl sulfoxide (DMSO) or untreated controls. These results demonstrate that curcumin disrupts the secretion of both SPATEs (Pet and EspC) in a concentration- and time-dependent manner.

### Curcumin Blocks the Secretion of SPATEs Without Affecting the Gene Expression of *pet* and *espC*

**Figure 3:**
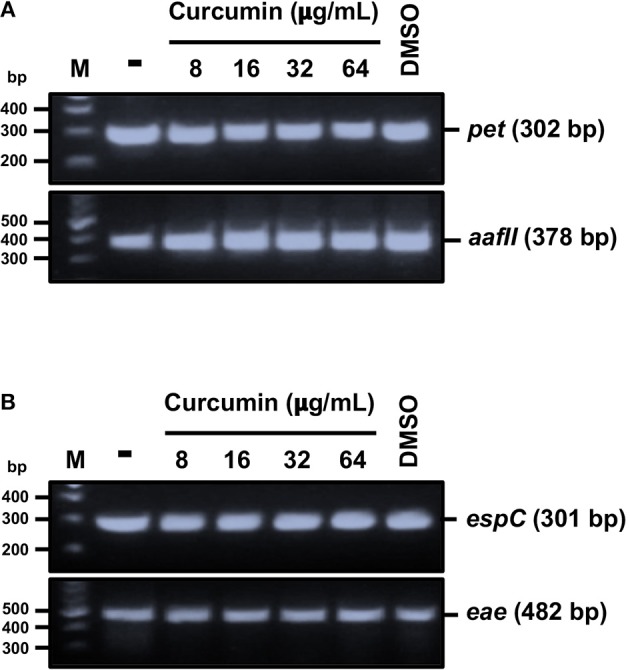
Curcumin does not alter both *pet* and *espC* gene expression. Total RNA was isolated from bacteria (EAEC or EPEC) in the presence or absence of curcumin, as described in Figure 2. RT-PCR assay and amplicons were analyzed by agarose gel electrophoresis (described in section Materials and Methods). Analysis of gene expression of *pet* and *aafII* from EAEC **(A)**; *espC* and *eae* from EPEC **(B)**. As controls, bacteria without curcumin or vehicle only (DMSO) were used.

In order to discern if the expression of SPATEs were affected by curcumin, we evaluated the expression of *pet* and *espC* genes after 2 h of curcumin treatment. Total mRNA from curcumin-treated bacteria was isolated, and the expression of *pet* and *espC* was analyzed by RT-PCR (Figure 3). The expression of *aafII* (Figure 3A) and *eae* (Figure 3B) mRNA from EAEC and EPEC, respectively, were used as positive controls. There were no notable differences between untreated bacteria and curcumin-treated bacteria (EAEC or EPEC) in both *pet* and *espC* mRNA expression (Figures 3A,B). Therefore, these results indicate that the inhibition of SPATEs secretion was not due to differences in mRNA expression.

### Curcumin Prevents the Releasing of Pet and EspC From the Bacterial Outer Membrane

**Figure 4:**
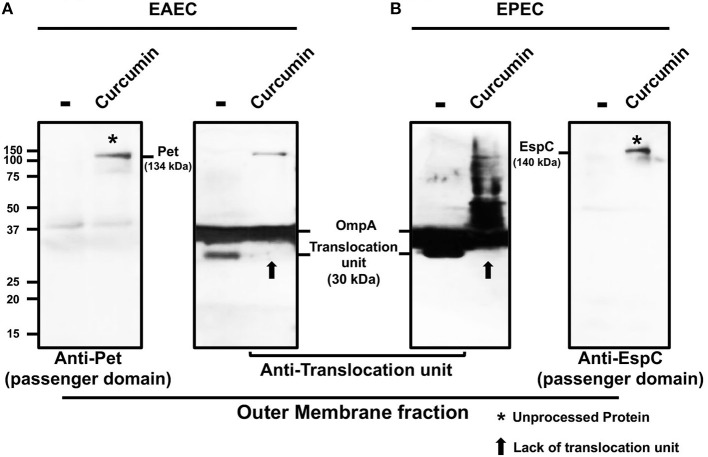
Pet and EspC toxins remained anchored in the outer membrane in the presence of curcumin. Bacterial strains EAEC 042 and EPEC E2348/69 were incubated with or without curcumin (16 μg/mL) for 2 h at 37°C at 150 rpm. Bacterial strains were fractionated into periplasmic and OM as described in section Materials and Methods. The proteins were analyzed by Western-blot using specific primary antibodies against Pet, EspC, translocation unit (β-domain), followed by an HRP-conjugated rabbit anti-mouse IgG2a secondary antibody. Outer membranes from treated and untreated EAEC **(A)** and EPEC **(B)** show processed and unprocessed Pet and EspC, respectively. Unprocessed protein (134 kDa for Pet and 140 kDa for EspC) consists of the passenger domain together with the translocation unit treated with curcumin (*). Arrows indicate the absence of the translocation unit (β-domain) in presence of curcumin (**↑**).

To further examine the molecular mechanisms by which curcumin inhibits the secretion of SPATEs, we evaluated the processing of Pet and EspC from the bacterial outer membrane (OM). The autotransporter structure is composed of three major domains that contribute to its secretion; (i) a cleavable N-terminal signal sequence that directs the protein via a Sec-dependent mechanism into the periplasm, (ii) a passenger domain containing the effector function of the protein, and (iii) a C-terminal translocation domain which forms a β-barrel pore in the OM that facilitates passenger domain export to the extracellular milieu (Henderson et al., 2004). To determine whether curcumin had an effect on the processing of Pet and EspC, we fractionated the different bacterial compartments (cytoplasm, periplasm, and outer membrane) of EAEC and EPEC. Western blot analysis of the different fractions confirmed the presence of specific proteins, GroEL (cytoplasmic), β-lactamase (periplasm), and the translocation domain (β-barrel, outer membrane) from each cellular fraction as an internal control of cell fractionation (Supplementary Figure 1). The corresponding molecular weights of full-length and unprocessed Pet and EspC corresponded to the molecular weights of 140 and 145 kDa, respectively. Cleavage of the signal sequence in these proteins generates a 134 (Pet) and a 140 kDa (EspC) intermediate consisting of the passenger domain and the translocation domain (β-barrel). Proteolytic cleavage of this intermediate on the extracellular face of the OM releases the mature passenger domain to the medium as a 104 kDa protein (Pet) for EAEC and 110 kDa (EspC) for EPEC, while the translocation domain (β-barrel) remains embedded in the OM as a 30 kDa protein. The outer membrane fraction from both curcumin-treated EAEC and EPEC revealed a high-molecular-mass protein of Pet (134 kDa) (Figure 4A) and EspC (140 kDa) (Figure 4B), representing the unprocessed and anchored proteins in the OM (passenger domain + translocation domain). These data indicate that curcumin blocked the release of mature Pet and EspC proteins from the OM into the extracellular milieu.

To further analyze the inhibition of SPATE secretion, we evaluated the proteolytic cleavage of the translocation domain (β-barrel) in untreated and curcumin-treated EAEC and EPEC; this domain remains in the OM as a 30 kDa protein upon release of the passenger domain. OM fractions from untreated and treated-bacteria were probed with an antibody that recognizes both of the translocation domain (β-barrel). The translocation domains (β-barrel) for both EspC and Pet share ~90% sequence homology; however, antibodies with reactivity against both proteins also have cross-reactivity with OmpA protein, a β-barrel anchored in the OM (Tapia-Pastrana et al., 2012). The translocation domain (β-barrel) of approximately 30 kDa was detected in the OM fraction from both untreated EAEC and EPEC (Figures 4A,B). While curcumin treatment inhibited the cleavage between the passenger and the translocation domains (β-barrel), this 30 kDa protein was not detected in the OM fractions of both EAEC and EPEC, and remained associated to the passenger domain as unprocessed proteins of higher molecular weights, i.e., 134 and 140 kDa, respectively (Figures 4A,B). These results suggest that curcumin can inhibit the proteolytic cleavage of SPATEs from the translocation domain (β-barrel) in the OM, keeping Pet and EspC anchored in the OM as unprocessed proteins (passenger domain + translocation domain), thereby blocking their release into to the extracellular milieu.

### Curcumin Inhibits the Cytotoxic Effect of EAEC and EPEC on HEp-2 Cells

**Figure 5:**
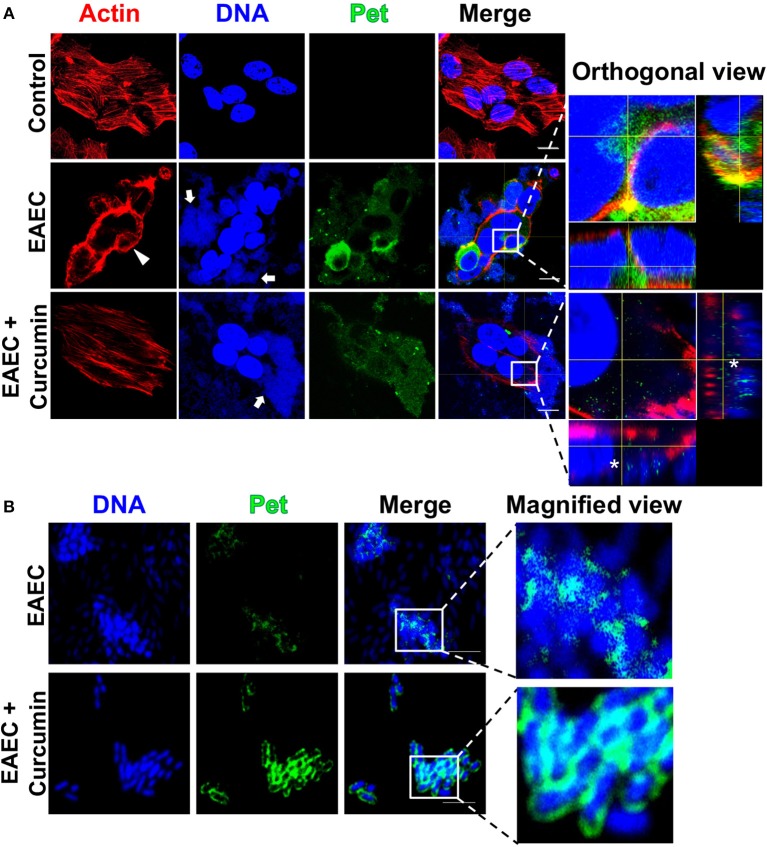
Curcumin blocks the cytotoxic effect induced by EAEC (rounding cells) in HEp-2 cells by inhibiting Pet internalization without affecting bacterial adherence to epithelial cells. Confocal microscopy of HEp-2 cells infected with EAEC (MOI 10) during 2 h **(A)** and a magnified view of bacteria-only **(B)** in the presence or absence of Curcumin (16 μg/mL). Uninfected cells were used as control. After infection, cells were washed, fixed, permeabilized, and stained with rhodamine-phalloidin for actin filaments and TO-PRO-3 for DNA detection. Pet secretion and internalization was detected by immunofluorescence using an anti-Pet passenger domain antibody followed by biotin-SP-conjugated goat anti-rabbit IgG and DTAF-conjugated streptavidin. The preparations were analyzed and documented with a confocal microscope (63×). Middle sections of the three channels were analyzed in *z*-stack, and the representative orthogonal views are shown with a higher magnification. Bacteria-only in the presence of curcumin are washed then stained for Pet as described above. A magnified view of a merge image is shown. Arrows indicate the aggregative adhesion pattern of EAEC on HEp-2 cells. Arrowheads indicates the cell rounding effect and an asterisk denotes the absence of Pet in HEp-2 cells; scale bar 15 μm in **(A)**. Scale bar 5 μm in **(B)**.

**Figure 6:**
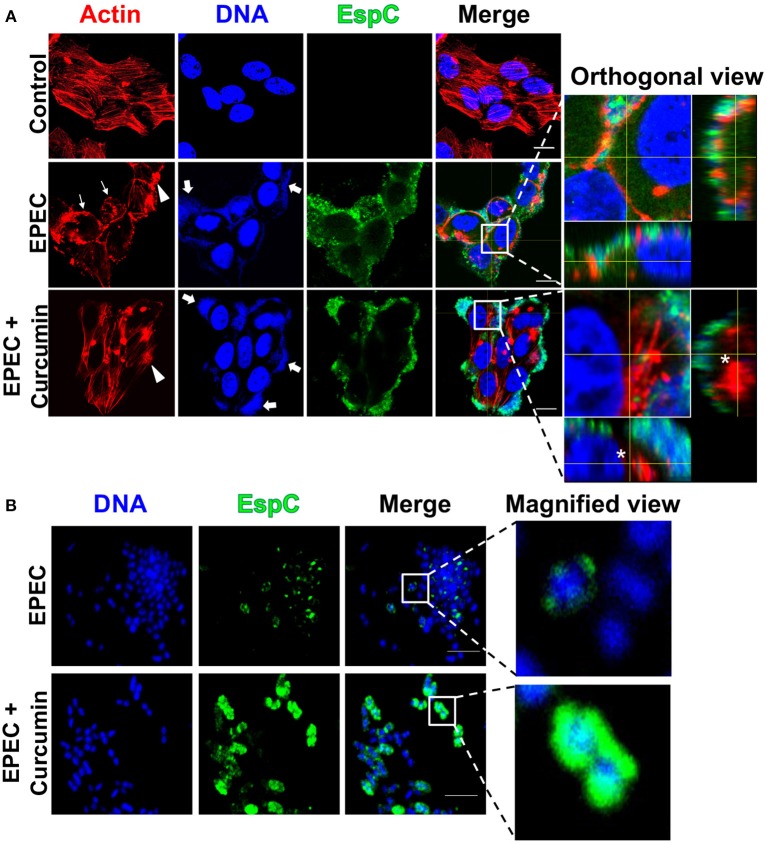
Curcumin blocks the cytotoxic effect induced by EPEC in HEp-2 cells by inhibiting EspC internalization without affecting the formation of pedestals and its adherence pattern on epithelial cells. Confocal microscopy of HEp-2 cells infected with EPEC (MOI 10) during 2 h **(A)** and a magnified view of bacteria-only **(B)** in presence or absence of Curcumin (16 μg/mL). Uninfected cells were used as control. After treatment, cells were processed for confocal microscopy as indicated in Figure 5, but EspC secretion and internalization was detected by immunofluorescence using an anti-EspC passenger domain antibody followed by biotin-SP-conjugated goat anti-rabbit IgG and DTAF-conjugated streptavidin. Arrow indicates the localized adherence of EPEC on HEp-2 cells. Arrowheads indicates the formation of actin pedestals, while thin arrows indicate the cell rounding effect. An asterisk denotes the absence of internalized EspC in HEp-2 cells; scale bar 15 μm in **(A)**. Scale bar 5 μm in **(B)**.

It has been shown that Pet and EspC autotransporter proteins have similar cytotoxic effects on epithelial cells during infection. Both of these toxins induce contraction of the actin cytoskeleton, loss of actin stress fibers, and release of focal contacts followed by complete cell rounding and detachment from the substratum (Navarro-Garcia et al., 2010). In order to evaluate if curcumin was capable of blocking the cytotoxic effects induced by Pet and EspC, secreted by EAEC and EPEC, respectively, we decided to investigate the role of curcumin treatment on infected HEp-2 cells with either EAEC or EPEC. Bacterial cultures of EAEC or EPEC were incubated for 2 h in the presence or absence of curcumin (16 μg/mL) and then used to infect HEp-2 cells (MOI 10) for an additional 2 h. Infected cells were prepared for confocal microscopy by staining the actin cytoskeleton with rhodamine-phalloidin (red), nuclear and bacterial DNA with TO-PRO-3 (blue), and Pet and EspC internalization using anti-Pet or anti-EspC (passenger domain) antibodies followed by a secondary antibody labeled with biotin-SP-conjugated and DTAF-conjugated streptavidin (green). In cells infected with EAEC, blue bacteria were observed to adhere in the classic aggregative adherence pattern, with the typical stacked-brick binding pattern (Figure 5A, arrows). The cytotoxic effects characterized by the loss of actin stress fibers and cell rounding were also observed (Figure 5A, arrowheads) and, as expected, Pet toxin was detected inside the cells as visualized by the orthogonal view of a middle z-section of infected cells (Figure 5A). However, curcumin-treated bacteria were not able to cause these cytotoxic effects, and it is possible to detect undisturbed actin stress fibers, similar in appearance to mock-treated cells (Figure 5A). Curcumin-treated EAEC still displays the classical aggregative adherence pattern in the absence of internalized Pet toxin, as visualized in the orthogonal view of a middle z-section (Figure 5A, asterisk). On the other hand, in cells infected with untreated-EPEC, it was possible to observe the characteristic localized adherence pattern forming bacterial microcolonies, and the accumulation of polymerized actin beneath the site of bacterial attachment forming pedestal-like structures (Figure 6A, arrows and thin arrows, respectively). These infected cells also displayed the classical cytotoxic effects, characterized by cytoskeleton contraction, cell rounding, and cell detachment, as previously reported (Navarro-Garcia et al., 2010) and EspC was localized inside the cells as visualized by the orthogonal view of a middle z-section of these infected cells. However, epithelial cells infected with curcumin-treated EPEC did not show these cytotoxic effects (cell rounding), although the formation of pedestals along the cells was not abrogated (Figure 6A, arrowheads). Similar to the observation with EAEC, in cells infected with curcumin-treated EPEC, EspC was not internalized in cells as observed in an orthogonal view of a middle z-section (Figure 6A, asterisk). Notably, the internalization of another effector molecule which also requires a T3SS, EspF, is not affected by the presence of curcumin (Supplementary Figure 2).

These data indicate that curcumin treatment inhibited the translocation of Pet and EspC into epithelial cells, as visualized in orthogonal views of a middle z-section of infected cells (Figures 5A, 6A, see orthogonal view, denoted by an asterisk). Both EAEC and EPEC maintain their adherence patterns, while EPEC is still able to form actin pedestal-like structures. These results suggest that curcumin has an important role in the inhibition of Pet and EspC secretion which controls the characteristic pathogenic mechanisms leading to the loss of cell integrity by both pathotypes on epithelial cells (Figures 5A, 6A, denoted by an arrowhead and thin arrows). However, the ability of curcumin-treated bacteria to maintain some of their pathogenic characteristics such as their adherence patterns (EAEC and EPEC) and the formation of pedestals (EPEC), suggests that other virulence factors involved in adhesion and the function of the T3SS are not affected by curcumin. Since curcumin treatment can block the secretion of SPATEs and inhibit their cytotoxicity on epithelial cells, we decided to investigate the localization of Pet and EspC by confocal microscopy over non-permeabilized bacteria to detect the presence of Pet or EspC on bacteria. Curcumin-treated bacteria showed a higher accumulation of Pet (Figure 5B) and EspC (Figure 6B) on their surface, as compared with untreated bacteria, in which no protein accumulation was observed. Altogether, these data suggest that curcumin prevents the release of Pet and EspC to the extracellular milieu promoting its accumulation on the bacterial surface, thereby avoiding the internalization on epithelial cells.

### Predicted Curcumin Binding Site on Pet and EspC

**Figure 7:**
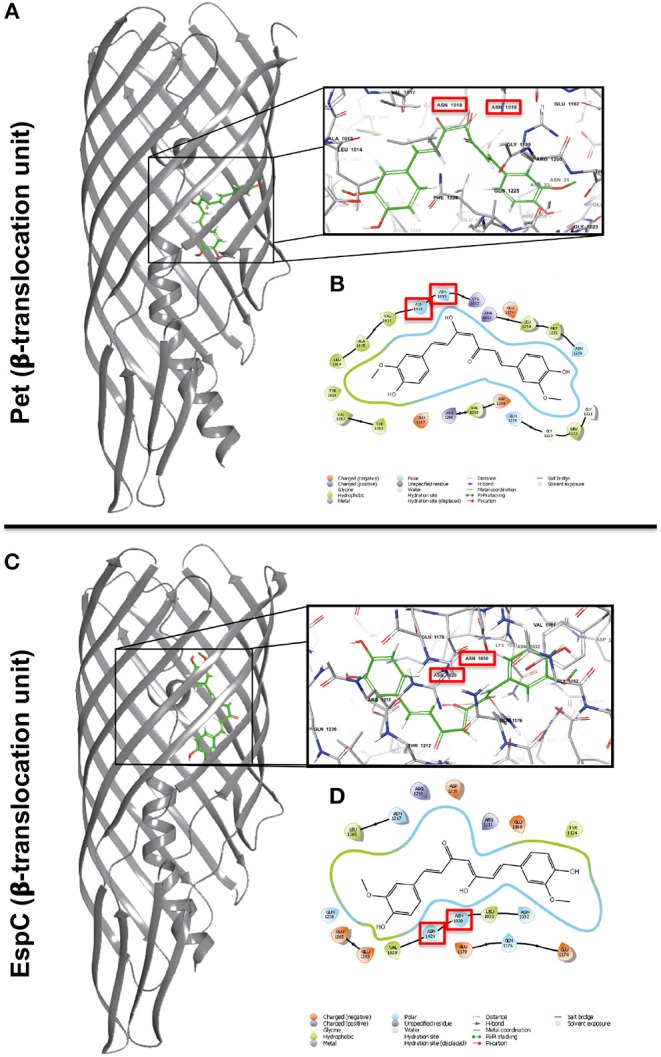
Representation of the Pet and EspC β-barrel translocation units and the predicted binding modes of curcumin to the protein. Predicted binding modes of curcumin at the Pet **(A)** and EspC **(C)** β-barrel translocation units. Most probable binding mode calculated for curcumin as predicted by AutoDock at the Pet and EspC β-domains, respectively **(B,D)**. Red rectangle highlights the interaction of important asparagine residues required for the release of Pet and EspC β-barrels.

Previously, it has been shown that a mutation in the predicted cleavage site N_1018_-N_1019_ of Pet inhibits the release of mature Pet from the translocation domain (β-barrel) (Navarro-Garcia et al., 2001). This effect has also been shown in another SPATE protein (EspP from EHEC), in which an aspartate within the β-barrel (D_1120_) and an asparagine (N_1023_) at the P1 position of the cleavage site, are essential for passenger domain cleavage. These two conserved residues are essential for the proteolytic processing mechanism, which form a unique catalytic dyad that mediates self-cleavage through asparagine cyclization (Dautin et al., 2007). In order to evaluate whether curcumin interacted with the translocation domain of Pet and EspC, we carried out molecular docking simulations. We generated homology models of Pet and EspC translocation domains (β-barrel) employing the EspP (PDB 3SLJ) crystallographic three-dimensional (3D) structure as a template. Using a sequence alignment tool, we compared the sequence conservation of the translocation domain (β-barrel) of Pet and EspC, with respect to EspP. Both Pet and EspC show high sequence conservation as well as the presence of essential residues involved in autocatalytic cleavage (Supplementary Figure 3). The Ramachandran plots of EspC and Pet confirm that both models are characterized by stereochemical parameters of a stable structure, with more than 90% residues falling in the most favorable regions. Additionally, the QMEAN6score show acceptable values that fall within an expected range for high-resolution crystal structures, (−0.02 for Pet and −0.330 for EspC) (Supplementary Figures 4A,D). The model structures were also validated by VERIFY-3D, with 73.15 and 75.83% of the amino acids on Pet and EspC, respectively, and an average 3D-1D score >0.2, indicating the reliability of the proposed model. Molecular docking modeling of curcumin on Pet (Figure 7A) or EspC (Figure 7C) was performed by Autodock using two boxes; an outer box covering all the Pet and EspC translocation domains (β-barrel), and an inner box, showing the site with the highest probability of binding in the interface of asparagine active-site. The docking score of the Pet or EspC and curcumin (−7.82, and −9.32, respectively) showed that curcumin had a perfect fit (Supplementary Table 1). Consequently, based on the interaction energies, a set of energetically favorable binding sites were identified. These results demonstrated that curcumin is positioned in the translocation domain (β-barrel), mainly where residues N_1018−1019_ and E_1168_ of Pet (Figures 7A,B), and N_1029−1030_ and E_1161_,_1179_ from EspC (Figures 7C,D) are located. As previously shown, these asparagine residues mediate the cleavage of both SPATEs, and glutamic acid is important considering that mutations introduced in EspP at position E_1172_ and E_1154_ impaired the cleavage of the passenger domain (Dautin et al., 2007). We also observed interactions with the amino acid residues in the same position for both translocation domains of Pet (V_1017_, R_1201_, D_1205_, Q_1226_, E_1275_) and EspC (V_1028_, R_1212_, D_1216_, Q_1237_, E_1286_). Although both translocation domains are highly conserved, interactions were observed with different residues for Pet (L_1014_, A_1015_, K_1022_, T_1044_, G_1222_, L_1223_, G_1224_, R_1253_, L_1255_, M_1256_, S_1257_, Y_1282_, V_1284_) and EspC (L_1031_, N_1032_, Y_1125_, E_1175_, Q_1177_, E_1284_) (Figure 7). The observed differences in amino acid binding between Pet and EspC are not clear could be due to inability of the predictive models to use dynamic structures in their analysis. Nonetheless, the differences in amino acid interactions of the translocation domains (β-barrel) of Pet and EspC, or residues outside of the cleavage site with curcumin, could also be directly or indirectly playing a role in the inhibition of toxin release. The use of rigid structures to predict binding does not take conformational changes in account as part of the analysis. Nonetheless, the predicted interaction of curcumin with the translocation domains (β-barrel) of both Pet and EspC provides an explanation for their accumulation on the bacterial outer membrane, and as a consequence the blocking of their characteristic pathogenic effects on epithelial cells.

## Discussion

The extensive use of antibiotics in recent decades against pathogenic bacteria such as *E. coli, Klebsiella pneumoniae, Staphylococcus aureus, Streptococcus pneumoniae*, and different *Salmonella* spp. has intensified antibiotic resistance among these species. Therefore, this challenge has resulted in a global health concern for its potential to increase the difficulty in treating diseases caused by such bacterial pathogens. One approach to combat this threat is the development of natural compounds with antibacterial properties as a potential therapy, or for the potentiation of commonly-used antibiotics. The present study evaluated the effect of curcumin on the cytotoxic effects induced by two serine protease autotransporter of *Enterobacteriaceae* (SPATEs) proteins, Pet and EspC, which belong to class I of SPATEs. Both Pet and EspC play a fundamental role in the pathogenesis of EAEC and EPEC, respectively. Curcumin treatment inhibited the proteolytic cleavage of Pet and EspC by preventing the release of the passenger domain from the OM, causing the accumulation of these proteins on the bacterial surface. This mechanism blocks the delivery of Pet or EspC into the extracellular milieu, thereby affecting their internalization into epithelial cells. Lack of Pet and EspC in the bacterial culture supernatants of curcumin-treated EAEC and EPEC was not related to an alteration in gene expression of these SPATEs. Additionally, curcumin had no bactericidal effects on EAEC or EPEC, since incubation of these bacteria with curcumin did not affect bacterial growth.

Despite their structural similarities, Pet and EspC use different mechanisms to gain access into the cytoplasm of a host cell. Nevertheless, they induce very similar cytopathic effects, causing the contraction of the host cell cytoskeleton, cell rounding, and subsequent detachment from the substratum (Navarro-Garcia et al., 1999, 2004). Pretreatment of bacteria with curcumin was capable of blocking the translocation of Pet and EspC into the cytoplasm of infected epithelial cells, thereby inhibiting the contraction of the cell cytoskeleton. However, curcumin treatment did not affect the characteristic adherence pattern associated with each pathotype (aggregative, EAEC; localized, EPEC), or the formation of actin pedestals by EPEC infection. These results strongly suggest that curcumin does not have a bactericidal effect in this model. Previously, it has been demonstrated that the internalization of Pet in epithelial cells is mediated by receptor-mediated endocytosis, whereas EspC requires a type III secretion system (T3SS) (Navarro-Garcia et al., 2004; Vidal and Navarro-Garcia, 2008). Interestingly, the presence of curcumin blocked the internalization of EspC without affecting the function of the T3SS, as demonstrated by the formation of actin pedestals and internalization of EspF, a T3SS effector protein, in the presence of curcumin. Previous analysis demonstrated that curcumin treatment affected the motility function, not the level of gene expression, of the T3SS, which forms the flagellar machinery of *S. enterica* serovar Typhimurium (class I and III) (Marathe et al., 2016). The accumulation of Pet and EspC on the bacterial membrane, strongly suggest that curcumin affects the secretion of Pet and EspC by affecting its release and the subsequent internalization into epithelial cells.

Recent studies have described the antibacterial properties of curcumin to be within a range of 16–1,000 μg/mL against a broad range of bacteria, including Gram-negative bacteria such as *E. coli* (Moghadamtousi et al., 2014). In this study, different concentrations of curcumin were utilized (up to 64 μg/mL) to determine if curcumin affected the growth of either EPEC or EAEC. Nonetheless, curcumin treatment was incapable of affecting the growth of EPEC and EAEC at 4 h. Similar observations have been reported previously for uropathogenic *E. coli* and *S. enterica* serovar Typhimurium using concentrations of curcumin of 100 μg/mL and 120 μM, respectively, without affecting bacterial growth (Packiavathy et al., 2014; Marathe et al., 2016). However, these studies demonstrated that curcumin affected important pathogenic processes, including biofilm formation (Packiavathy et al., 2014), and flagellum function (Marathe et al., 2016). In this context, we evaluated the role of curcumin in the secretion of Pet and EspC, which are key virulence factors in the pathogenesis of *E. coli*. Curcumin treatment efficiently inhibited the secretion of Pet and EspC into the extracellular milieu in a dose dependent manner, without affecting the genetic expression of *pet* and *espC*. However, the presence of Pet, and to some extent, EspC, in the supernatants of curcumin-treated bacteria at later time points could be due to an insufficient concentration of curcumin to block the secretion of SPATEs once a saturation phase was reached. These results, together with the detection of these proteins by confocal microscopy on the bacteria surface, suggest that curcumin had an effect on a specific process during the proteolytic processing mechanism which allows the SPATEs to be liberated into the extracellular milieu. To mediate their secretion, SPATEs contain three functional domains: an N-terminal sequence, an extracellular passenger domain, and a translocation domain (β-barrel) on the C-terminus (Dautin, 2010). The translocation units (30 kDa, β-barrel domain) of Pet and EspC form a pore on the outer membrane of EAEC and EPEC, respectively, through which the passenger domain (104 kDa of Pet or 110 kDa of EspC) is translocated on the bacterial surface. This passenger domain is liberated into the extracellular milieu by a proteolytic mechanism independent from its serine-protease domain (GDSG) (Navarro-Garcia et al., 2010). Remarkably, curcumin was capable of blocking the proteolytic cleavage between the passenger domain and the translocation unit of Pet and EspC on the outer membrane of EAEC and EPEC, respectively, and both were detected as 134 and 140 kDa proteins in this membrane fraction. These analyses correlated with the confocal microscopy evidence where Pet and EspC were seen decorating the bacterial outer membranes of curcumin-treated EAEC and EPEC. Members of the SPATE family contain a single cleavage site between the passenger and the translocation domains (β-barrel). The precise location of the proteolytic processing occurs between two asparagine (N) residues, which are highly conserved (EVNNLN) in class I of SPATEs. The proteolytic cleavage of Pet (104 kDa) occurs between N_1018_ and N_1019_ (Eslava et al., 1998), and in EspC (110 kDa) between N_1029_ and N_1030_ (Stein et al., 1996). It has been demonstrated that a mutation in the cleavage side of Pet, between amino acids N_1018_ and N_1019_, in which the asparagine residues were substituted for glycine and isoleucine, respectively, blocked the release of Pet and the protein remained anchored in the bacterial outer membrane as a 134 kDa complex (104 kDa passenger domain + 30 kDa translocation domain) (Navarro-Garcia et al., 2001). In fact, in curcumin-treated EAEC, unprocessed Pet was seen anchored on the OM, similar to a previous report for a *pet* mutant in the two asparagines (N_1018_ and N_1019_) (Navarro-Garcia et al., 2001). These data indicate a potential mechanism by which curcumin acts to block the processing and release of Pet and EspC to the extracellular milieu.

Previous studies have demonstrated that the highly conserved residues aspartic acid (D_1120_) and asparagine (N_1023_) form a unique catalytic dyad which facilitates the autoproteolytic cleavage mediated by the cyclization of the asparagines residues N_1023_-N_1024_, releasing the passenger domain of EspP (Dautin et al., 2007). The sequence alignment of the translocation domains (β-barrel) of EspP, Pet, and EspC indicate a high degree of conservation with ≥92% positive hits, and ≥81% identity (Supplementary Figure 3A). The residues N-N where the cleavage between the passenger and the β-barrel domains take place are highly conserved, and they are found in a site inaccessible to outer membrane or periplasmic proteases; therefore, Pet and EspC could also be processed by a similar autoproteolytic mechanism proposed for EspP. This suggests that the possible mechanism by which curcumin blocks the secretion of Pet and EspC occurs inside the translocation domain (β-barrel). Using computational docking modeling, we showed that the interaction of curcumin with the translocation domain occurs at the site where the residues N_1018_-N_1019_ of Pet and residues N_1029_-N_1030_ of EspC are located. These results suggest that curcumin blocks the cleavage, and therefore, the release of both SPATEs. The interaction of curcumin with the asparagine residues strongly suggests a mechanism for blocking the autoproteolytic process of cyclization of the asparagine, similar to the one proposed for EspP. However, the interaction of curcumin with residues N_1018_-N_1019_ in Pet and N_1029_-N_1030_ in EspC could directly or indirectly contribute to the inhibition of toxin release; therefore, future studies should focus in determining the specific amino acid residues involved in this effect. These results also provide a molecular explanation for the blockage of Pet and EspC release in our model. Previous docking studies have demonstrated, in a similar fashion, that the interaction of curcumin with the amino acid sequence N-D-S-G of the flagellum affects the flagellum's density, and as a consequence, the pathogenicity of *S. enterica* serovar Typhimurium. Furthermore, due to the high sequence conservation at the cleavage site of SPATEs, our study proposes a molecular mechanism by which curcumin blocks the secretion of SPATEs, mediated by the interaction with asparagine residues N_1018_-N_1019_ of Pet and N_1029_-N_1030_ of EspC, and blocking a key step for the proteolytic processing and efficient secretion into the extracellular milieu. The SPATEs belonging in class I constitute a large family of proteins secreted by the distinct diarrheagenic *E. coli* pathotypes, *Shigella flexneri, Salmonella bongori*, and *Citrobacter*. The distinct toxins secreted by these bacteria show different cytotoxic activities, which depend on the passenger domain structure. Nonetheless, the translocation domain, as well as the active site, are formed by highly conserved sequences. Curcumin can be an important antibacterial compound for its potential to block the secretion of two important toxins such as Pet and EspC; more importantly, this study proposes a mechanism by which curcumin may hinder the secretion of other SPATE-containing bacteria. Future studies should aim at determining if curcumin has synergistic effects with antibiotics, which would be particularly useful in the design of new antimicrobial therapies.

## Data Availability Statement

All datasets generated for this study are included in the manuscript/Supplementary Files.

## Author Contributions

JS-V participated in the design of the study, cytotoxicity, confocal microscopy, samples processing, data analysis, and writing of the manuscript. FN-G participated in the design of the study, data analysis, manuscript writing, and final approval of the manuscript. AC-R participated in the design of the study. FG-G carried out the *in-silico* study and data analysis. DT was involved in the analysis and writing of this manuscript. GT-P participated design of the study, bacterial cell fraction, autotransporter proteolytic processing, data analysis, manuscript writing, and final approval of the manuscript. All authors read and approved the final manuscript.

### Conflict of Interest

The authors declare that the research was conducted in the absence of any commercial or financial relationships that could be construed as a potential conflict of interest.
