# Supplementary material for: Curcumin Blocks Cytotoxicity of Enteroaggregative and Enteropathogenic Escherichia coli by Blocking Pet and EspC Proteolytic Release From Bacterial Outer Membrane
Source: Front Cell Infect Microbiol. 2019 Sep 25;9:334. doi: 10.3389/fcimb.2019.00334 (PMC6798032; doi:10.3389/fcimb.2019.00334)
Supplement: Supplementary file 1 [file Data_Sheet_1.PDF]

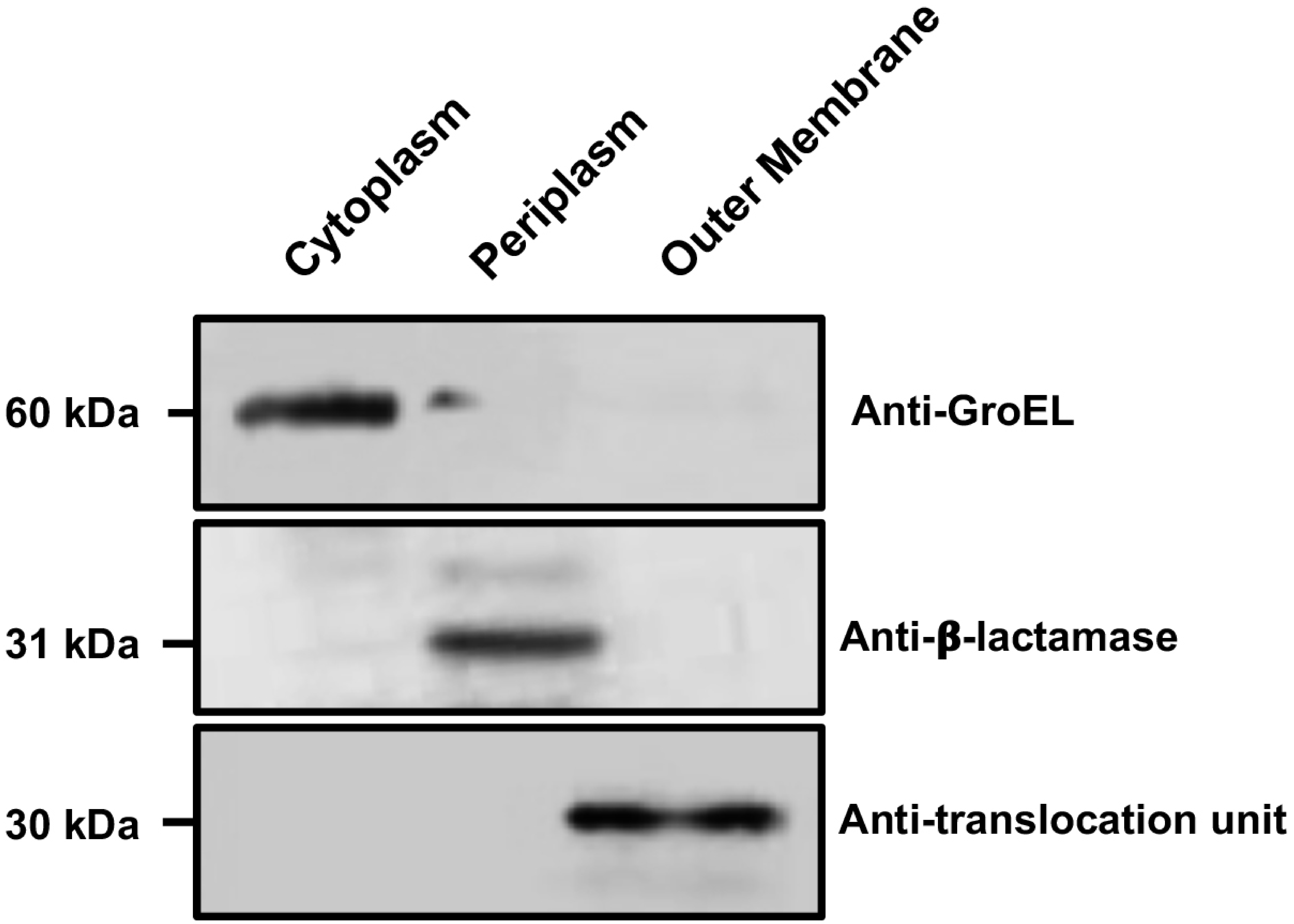

Supplementary Fig. 1. Validation of bacterial membrane fractionation during infection. Bacterial fractions extracted from EAEC were subjected to western analysis. Proteins from different fractions were analyzed using a specific primary antibody against GroEL (cytoplasmic fraction), β-lactamase (periplasmic fraction), and translocation unit (β-barrel) (outer membrane fraction), followed by an HRP-conjugated goat-anti rabbit IgG or an HRP-conjugated rabbit-anti mouse IgG2a secondary antibodies.

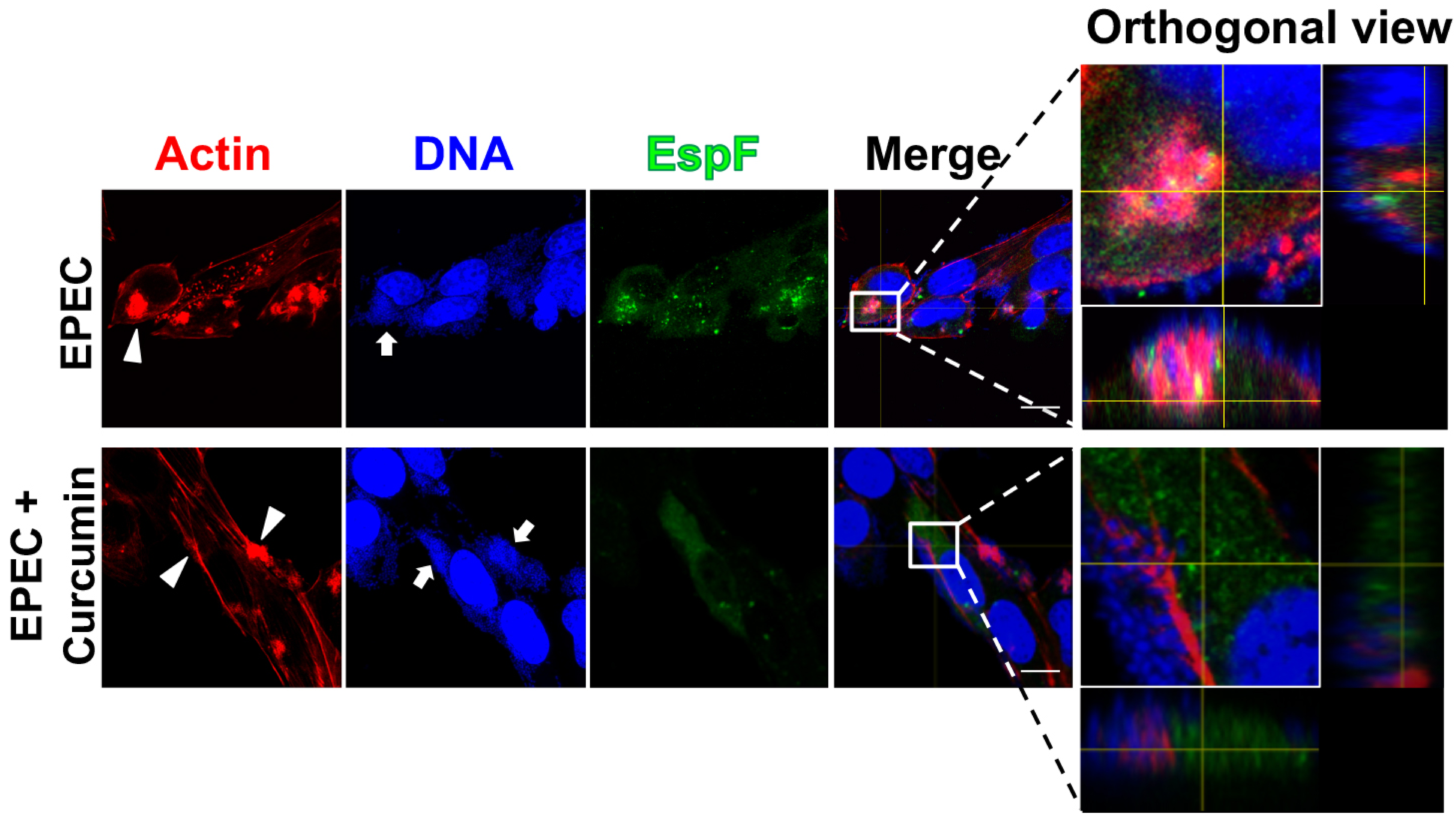

Supplementary Fig. 2. Curcumin does not affect the translocation of EspF from EPEC during infection, through the T3SS in HEp-2 cells, and the formation of actin pedestals. Confocal microscopy of HEp-2 cells infected with EPEC as described in Figure 5, but EspF internalization was detected by immunofluorescence with an anti-EspF antibodies followed by biotin-SP-conjugated goat-anti mouse IgG and DTAF-conjugated streptavidin. Scale bar 15 μm.

A

Alignment sequence analysis of β-barrel domains of Pet and EspC to the β-barrel sequence of EspP.

	% Identity with respect to EspP	% Similarity with respect to EspP
Pet*	88	95
EspC*	81	92

* β-barrel amino acid sequence domains were used in alignment analysis with respect to EspP

B

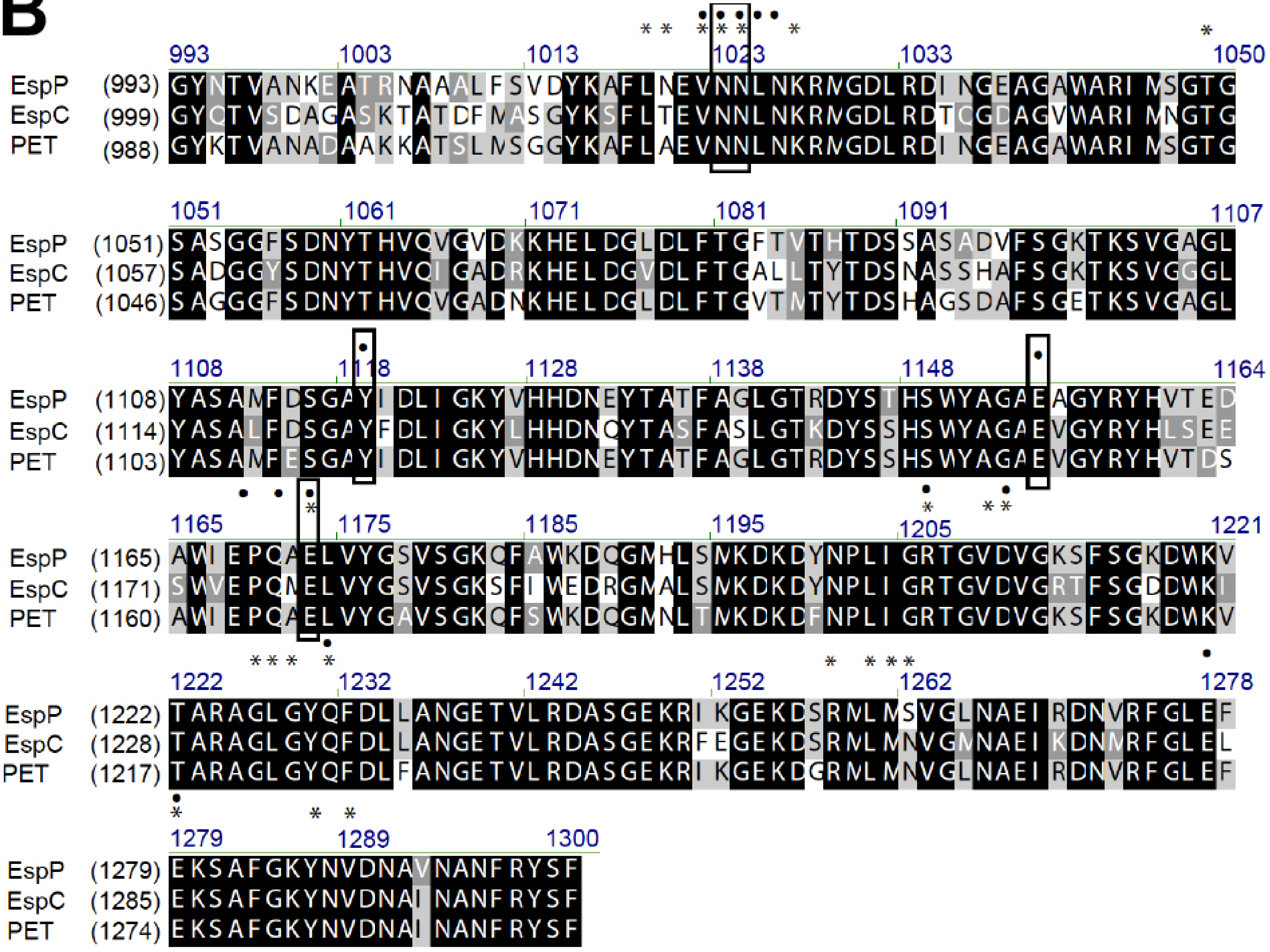

Supplementary Fig. 3. Alignment of the Pet (GenBank accession no. SJK83553.1), EspC (GenBank accession no. AAC44731.1), and EspP (GenBank accession no. OSJ99441.1) translocation domains (β-barrels). Residues labeled with a circle (•) highlight the residues involved in curcumin binding with the EspC translocation domain (β-barrel). Asterisks (*) labeled residues involved in curcumin binding with the Pet translocation domain (β-barrel), and box (□) denote residues involved in the active-site asparagine.

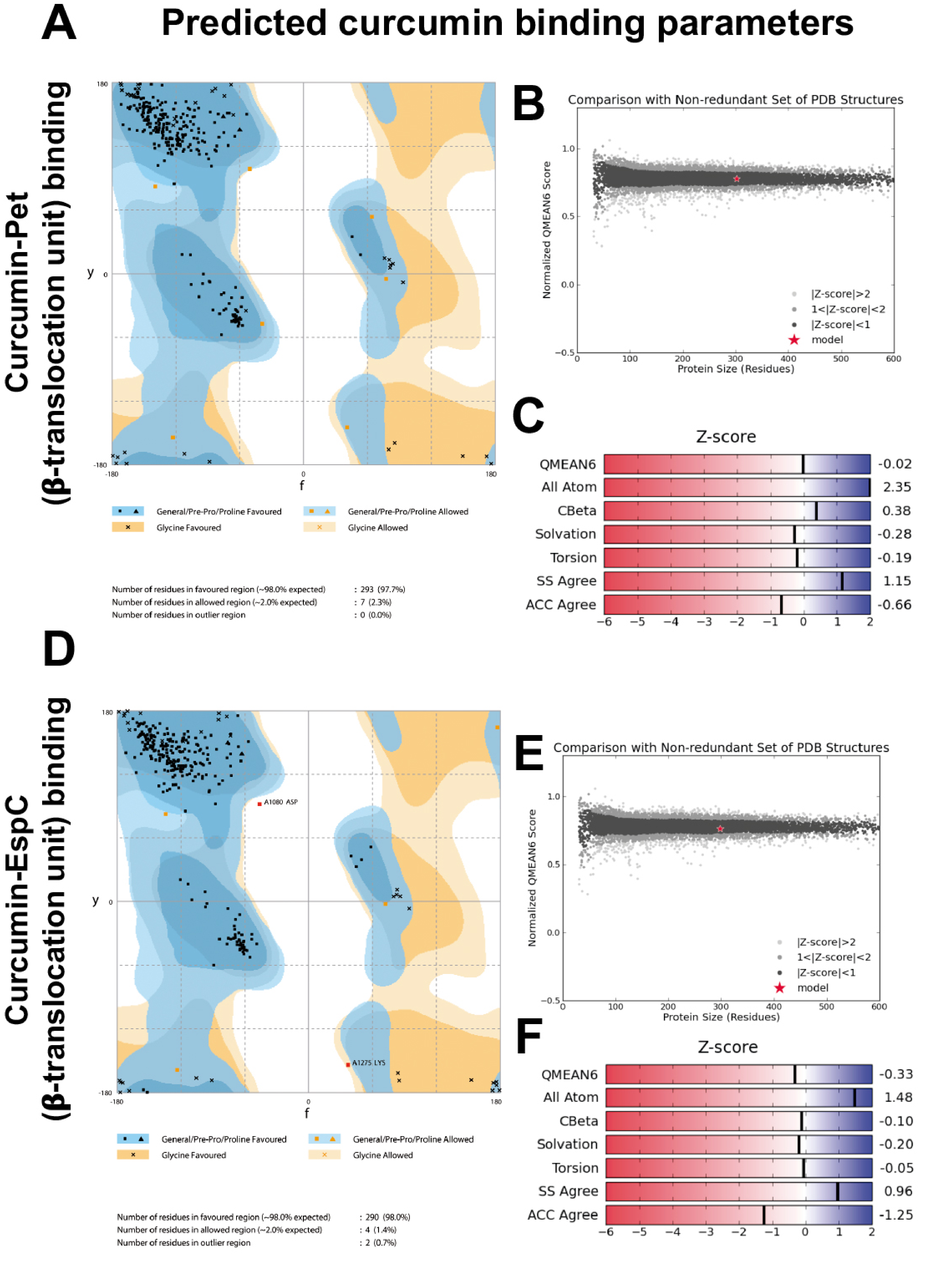

Supplementary Fig. 4. Structural validation of theoretical 3D structure for Pet and EspC β-Domains. Ramachandran plot analysis, 98% and 97.7% of total residues are in the most favored region for Pet and EspC, respectively **(A, D)**. Normalized QMEAN6 scores **(B, E)**, and Z-score diagrams of the individual components of QMEAN **(C, F)**.

Supplementary Table 1. Summary of docking score values (kcal/mol) per Pose and active residues

Pet		
**Cluster Number**	**Docking score (kcal/mol)**	**Amino Acids to 4 Å of curcumin**
1	-7.82	L 1014, A1015, V1017, N1018, N1019, K1022, Y1055, E1167, R1200, V1203, D1204, G1221, L1222, G1223, Q1225, R1252, L1254, M1255, N1256, E1274, Y1281, V1283
1	-7.71	K1011, A 1012, L 1014, A1015, V1017, N1018, N1019, K1022, D1051, D1053, Y1055, E1167, R1200, V1203, G1221, Q1225, R1252, L1254, M1255, N1256, E1274, Y1281, V1283
1	-7.59	K1011, A 1012, L 1014, A1015, V1017, N1018, N1019, D1053, Y1055, S1084, E1167, G1221, Q1225, R1252, L1254, M1255, N1256, E1274, Y1281, V1283
1	-7.57	L 1014, A1015, V1017, N1018, N1019, L1020, N1021, K1022, E1149, Y1055, Y1113, E1163, Q1165, E1167, R1200, R1219, G1221, L1222, G1223, Q1225, R1252, L1254, M1255, N1256, E1274, Y1281, V1283
1	-6.69	L 1014, A1015, V1017, N1018, N1019, K1022, Y1055, E1167, R1200, G1221, L1222, L1254, M1255, N1256, E1274, Y1281, V1283
1	-6.57	V1017, N1018, N1019, L1020, N1021, E1149, Y1055, Y1113, E1163, Q1165, E1167, R1200, R1219, G1221, L1222, G1223, Q1225, R1252, N1256, E1274, Y1281, V1283
2	-5.11	A1036, W1237, A1038, R1039, I1040, V1058, V1060, G1061, A1289, N1290, F1291
1	-5.01	A1015, V1017, N1018, N1019, K1022, Y1055, E1167, R1200, G1221, L1222, L1254, M1255, N1256, E1274, Y1281, V1283
2	-4.88	A1036, W1237, A1038, V1058, V1060, G1061, A1289, N1290, F1291
2	-4.08	W1237, A1038, R1039, I1040, V1058, V1060, G1061, A1289, N1290
**EspC**		
**Cluster Number**	**Docking score (kcal/mol)**	**Amino Acids to 4 Å of curcumin**
1	-9.32	V1028, N1029, N1030, L1031, N1032, Y1124, E1160, E1174, Q1176, E1178, R1211, D1215, R1230, Q1236, L1265, N1267, E1283, E1285
1	-9.25	L1025, V1028, N1029, N1030, L1031, N1032, K1033, E1160, E1174, Q 1176, E1178, R1211, V1214, D1215, G1232, L1233, G1234, Q1236, L1265, M1266, N1267, E1283, E1285, K1286
1	-7.79	N1029, N1030, L1031, N1032, K1033, G1036, D1037, L1118, E1160, R1164, E1174, Q1176, E1178, R1211, V1214, D1215, R1230, G1234, Q1236, L1265, M1266, N1267, E1283, E1285
1	-7.76	V,1028, N1029, N1030, L1031, N1032, K1033, G1036, D1037, Y1124, E1160, R1164, E1174, Q1176, E1178, R1211, V1214, D1215, R1230, Q1236, L1265, M1266, N1267, E1283, E1285
1	-7.49	V,1028, N1029, N1030, L1031, G1036, D1037, Y1124, E1160, R1164, E1174, Q1176, E1178, R1211, Q1236, L1265, M1266, N1267, E1283, E1285
1	-7.24	N1029, N1030, L1031, G1036, D1037, Y1124, E1160, R1164, E1174, Q1176, E1178, R1211, V1214, R1230, Q1236, M1266, N1267, E1283, E1285
1	-6.93	V1028, N1029, N1030, L1031, Y1124, E1160, E1174, Q1176, E1178, R1211, D1215, R1230, Q1236, N1267, E1283, E1285
1	-5.64	N1030, L1031, N1032, K1033, G1036, D1037, L1118, E1160, R1164, E1174, Q1176, E1178, R1211, G1234, Q1236, M1266, N1267, E1283, E1285
2	-3.63	V1161, G1162, Y1163, P1175, Q1176, M1177, T1212, G1213, V1214, L1233, G1234, Y1235
2	-3.10	V1161, G1162, Y1163, P1175, Q1176, M1177, T1212, G1213, V1214
